# Supplementary material for: Investigating the influence of physiologically relevant hydrostatic pressure on CHO cell batch culture
Source: Sci Rep. 2021 Jan 8;11:162. doi: 10.1038/s41598-020-80576-8 (PMC7794228; doi:10.1038/s41598-020-80576-8)
Supplement: Supplementary file 1 — Supplementary Information 1. [file 41598_2020_80576_MOESM1_ESM.zip › S1/IgG/Feb14 0 30 60 90/SS1 Feb8 2.pdf]

Data File C:\Users\P...emStation\1\Data\TK_CHO_IgG 2019-02-14 14-17-55\034-6-ML SS1 Feb8.D
Sample Name: ML SS1 Feb8

===

Acq. Operator : SYSTEM   Seq. Line : 34
Sample Operator : SYSTEM
Acq. Instrument : HPLC-0039   Location : 6
Injection Date : 2/14/2019 4:40:39 PM   Inj : 1
                                          Inj Volume : 100.000 µl
Method : C:\Users\Public\Documents\ChemStation\1\Data\TK_CHO_IgG 2019-02-14 14-17-55\CHO_IgG_LC.M (Sequence Method)
Last changed : 2/8/2019 3:17:23 PM by SYSTEM

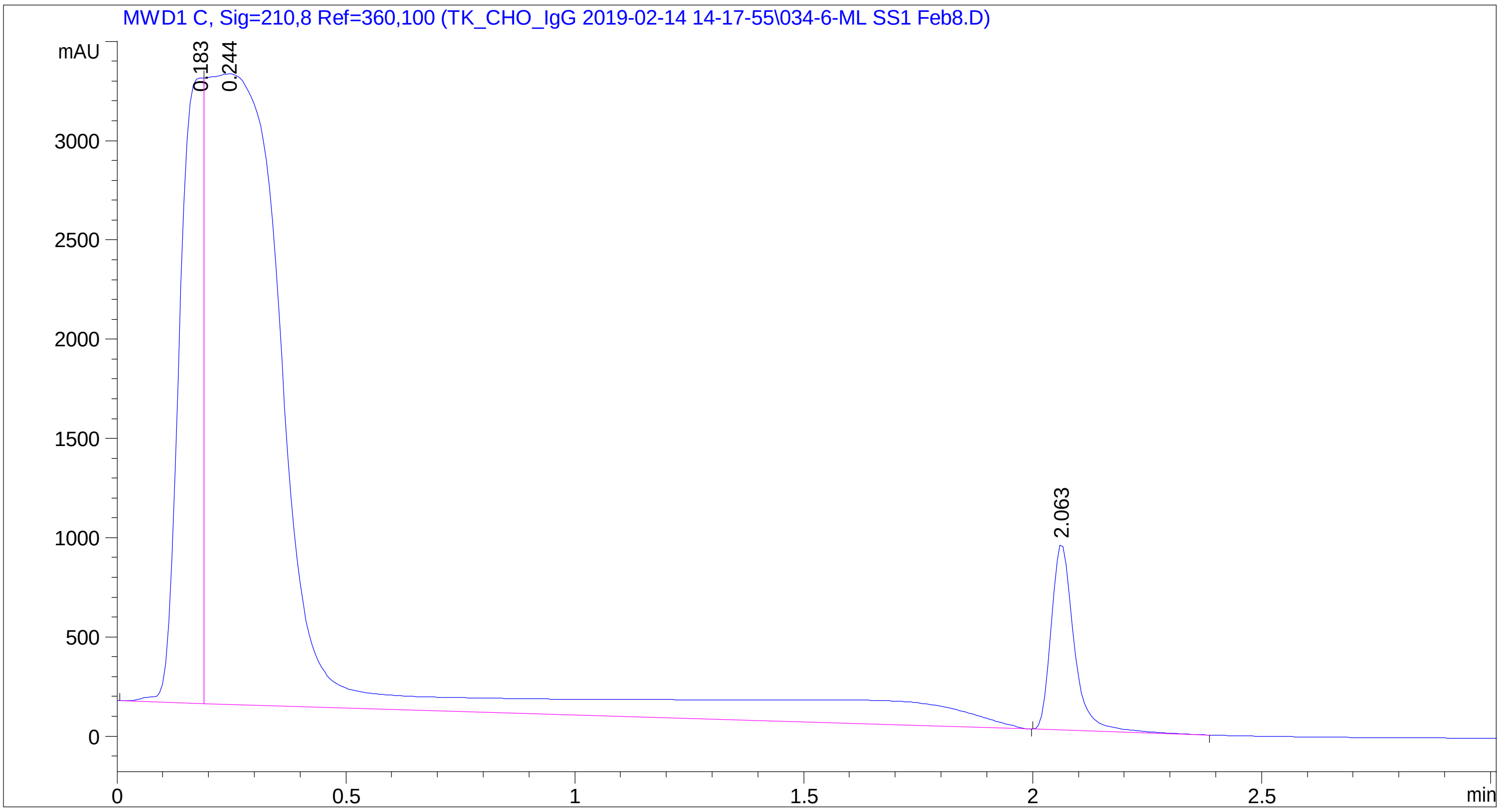

===

Area Percent Report

===

Sorted By : Signal
Multiplier : 1.0000
Dilution : 1.0000
Use Multiplier & Dilution Factor with ISTDs

Signal 1: MWD1 C, Sig=210,8 Ref=360,100

Peak #	RetTime [min]	Type	Width [min]	Area [mAU*s]	Height [mAU]	Area %
1	0.183	BV	0.0568	1.10148e4	3151.69458	19.6277
2	0.244	VB	0.1917	4.18380e4	3175.88184	74.5526
3	2.063	BBA	0.0546	3265.95776	938.72876	5.8197

Totals : 5.61188e4 7266.30518

===

*** End of Report ***
